# Supplementary material for: Stimulus-Responsive, Gelatin-Containing Supramolecular Nanofibers as Switchable 3D Microenvironments for Cells
Source: Polymers (Basel). 2022 Oct 19;14(20):4407. doi: 10.3390/polym14204407 (PMC9607093; doi:10.3390/polym14204407)
Supplement: Supplementary file 1 [file polymers-14-04407-s001.zip › polymers-1976274-SI.pdf]

Supplementary Materials to

"Stimulus Responsive, Gelatin-Containing Supramolecular Nanofibers as Switchable 3D Microenvironments for Cells"

Kentaro Hayashi, Mami Matsuda, Masaki Nakahata, Yoshinori Takashima, Motomu Tanaka

Supplementary Material S1. 2D ROESY NMR spectrum of amino-βCD/amino-Ad

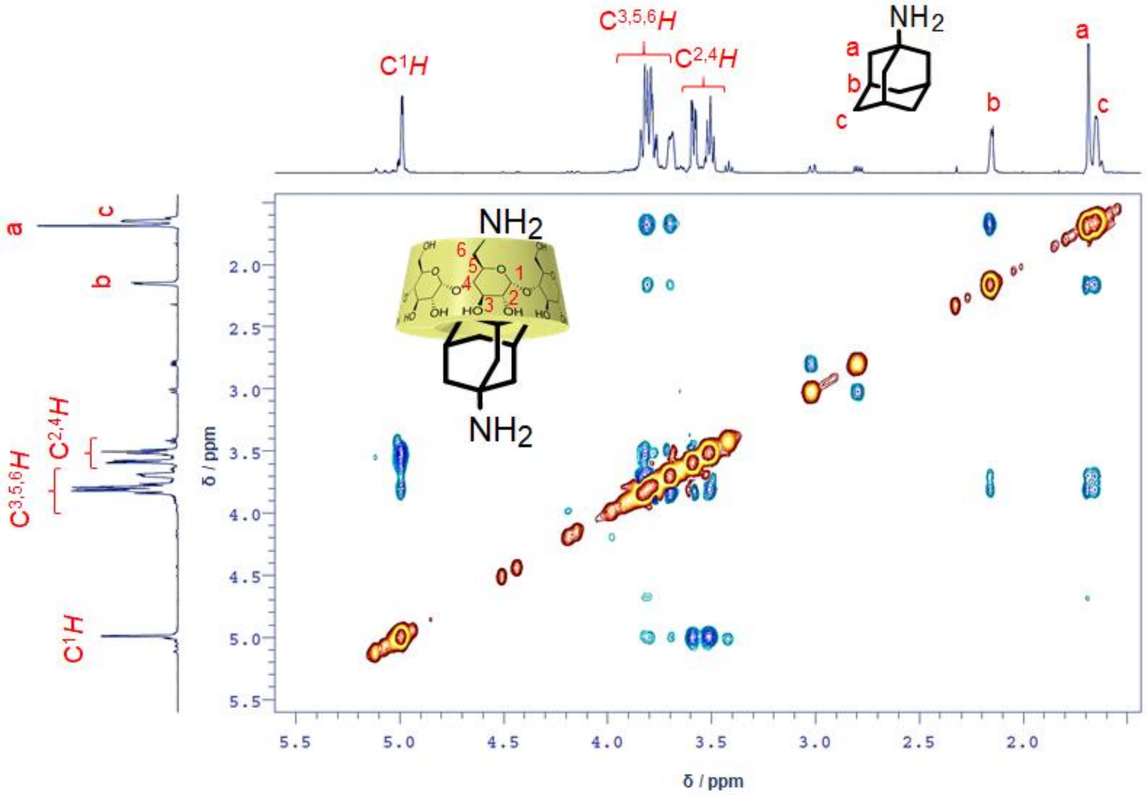

Figure S1. ^{1}H-^{1}H 2D rotating-frame nuclear Overhauser effect spectroscopy (ROESY) NMR spectrum of Amino-βCD/Amino-Ad complex in D_2O acquired on a 600 MHz JEOL ECA-600 NMR spectrometer at 25 °C. Chemical shifts were referenced to HOD as a standard (δ = 4.7 ppm). The peak of HOD was eliminated.

Supplementary Material S2. ATR-FTIR of materials related to gelatin-βCD-Ad (Method 1).

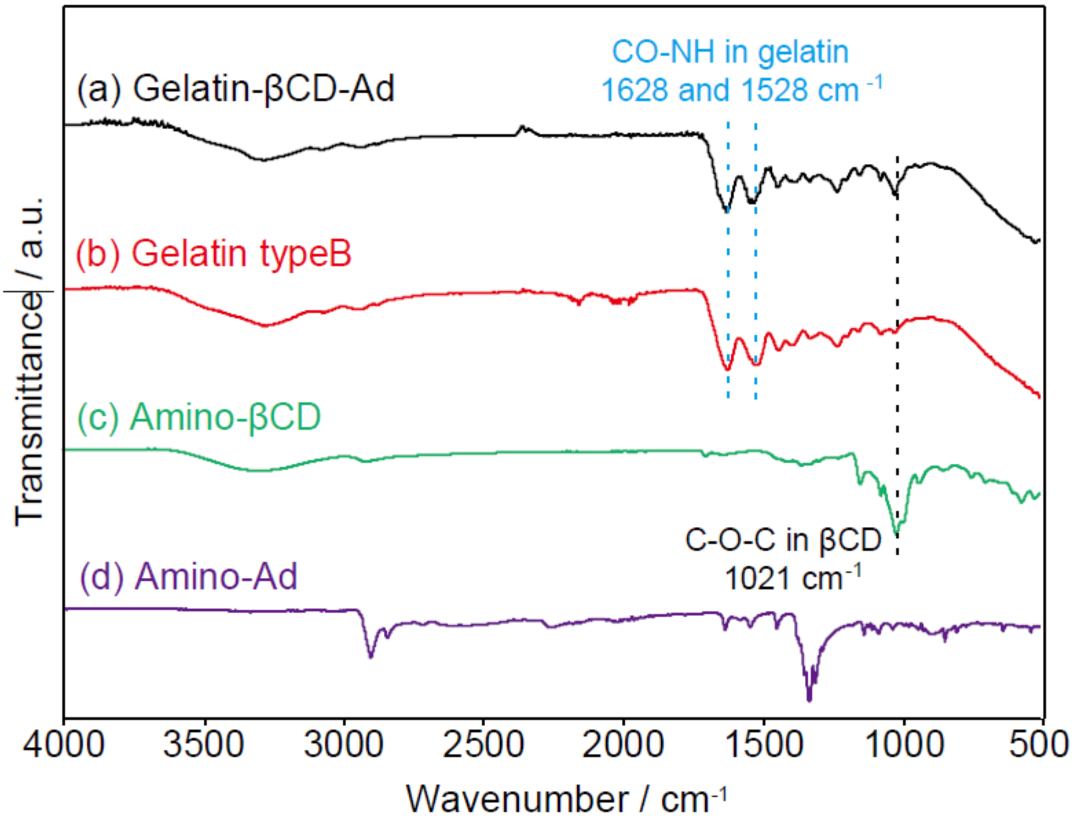

Figure S2. ATR-FTIR spectra of (a) gelatin-βCD-Ad, (b) gelatin type B, (c) amino-βCD, and (d) amino-Ad through diamond crystal acquired on a JASCO FT/IR 6100 spectrometer.

Supplementary Material S3. ATR-FTIR of materials related to gelatin-βCD-gelatin and Ad-gelatin (Method 2).

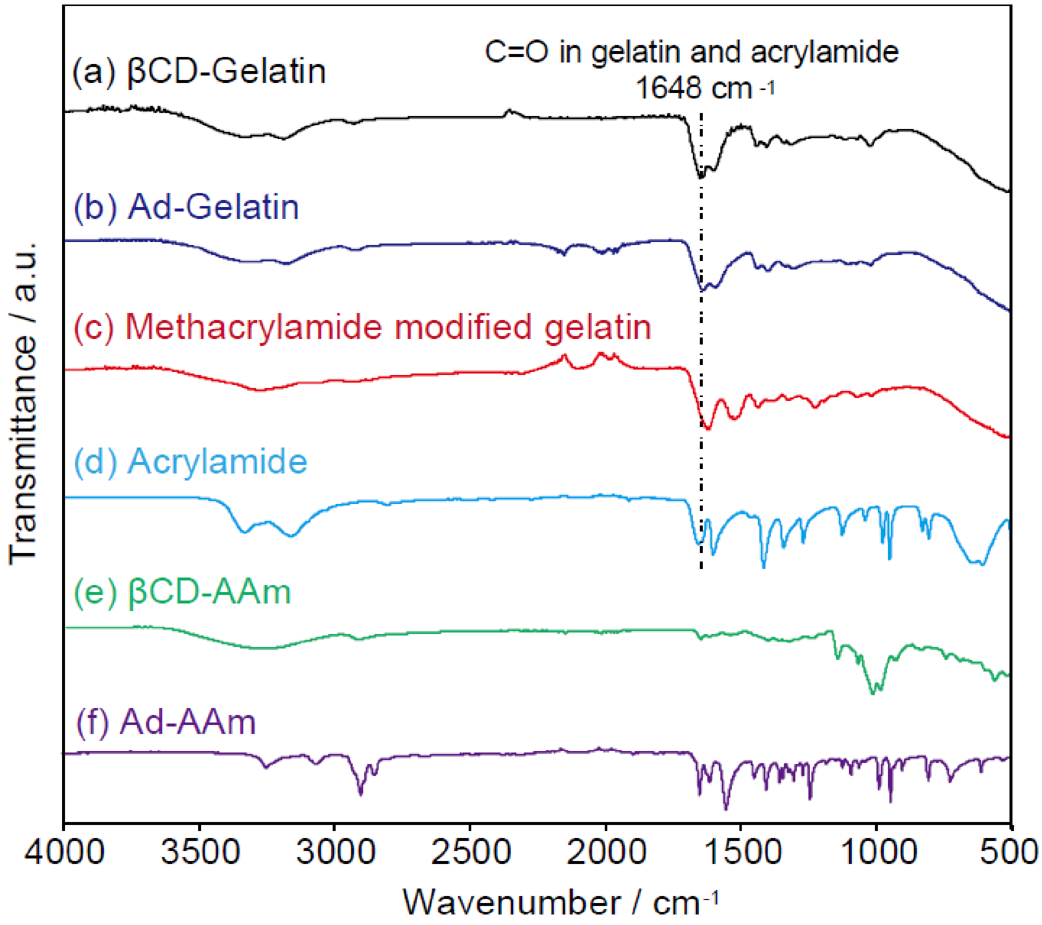

Figure S3. ATR-FTIR spectra of (a) βCD-gelatin, (b) Ad-gelatin, (c) methacrylamide modified gelatin, (d) acrylamide, (e) βCD-AAm, and (f) Ad-AAm through diamond crystal acquired on a JASCO FT/IR 6100 spectrometer.

Supplementary Material S4. Optical microscopy images of βCD-gelatin/ Ad-gelatin fibers (Method 2)

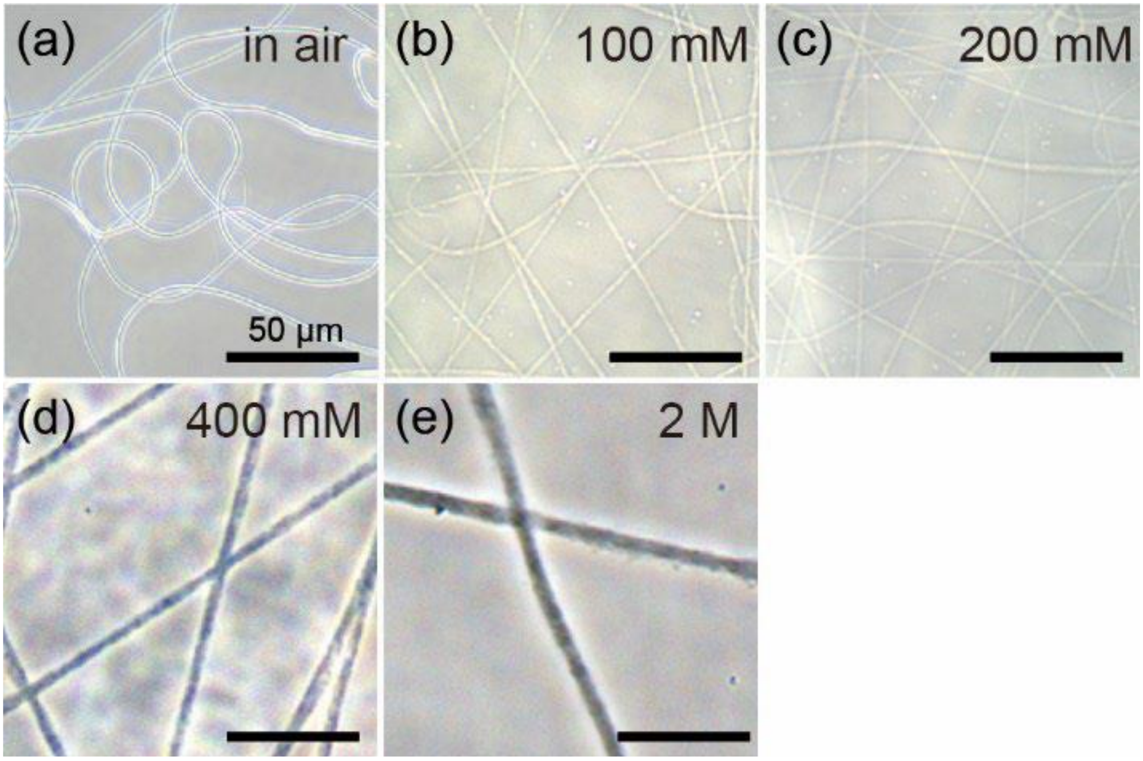

Figure S4. Optical microscopy images of βCD-gelatin/Ad-gelatin nanofibers **(a)** before chemical cross-linking (in air) and after cross-linking with **(b)** 100 mM, **(c)** 200 mM, **(d)** 400 mM and **(e)** 2 M NHS/EDC (in PBS).

Supplementary Material S5. Compositions of synthesized polymers.

Table S1 a. Composition of gelatin-βCD-Ad.

Gelatin	2.0 g
Carboxyl units in gelatin	2.2 mmol
Amino-βCD/amino-Ad	2.8 g / 2.2 mmol
EDC	1.5 g / 8.0 mmol
NHS	0.92 g / 8.0 mmol
MES buffer	40 mL

Table S1 b. Composition of βCD-gelatin.

AAm	0.7 g / 9.9 mmol
Methacrylamide-modified gelatin	25 mg
Methacryloyl units	3.3 μmol
βCD–AAm	0.12 g / 0.1 mmol
LAP	1.5 mg / 5 μmol
DMSO	5 mL

Table S1 c. Composition of Ad-gelatin.

AAm	0.7 g / 9.9 mmol
Methacrylamide-modified gelatin	25 mg
Methacryloyl units	3.3 μmol
Ad–AAm	21 mg / 0.1 mmol
LAP	1.5 mg / 5 μmol
DMSO	5 mL
